# Laryngology and Rhinology

**Published:** 1905-03

**Authors:** P. Watson Williams


### LARYNGOLOGY AND RHINOLOGY.

**Vincent's Angina.**—Bacteriological investigation has added to the number of cases which must be diagnosed as diphtheria, but it has also served to differentiate conditions which clinically are hardly distinguishable from the milder forms of the more formidable disease. Of these one which is occasionally met with is the diphtheroid angina due to the fusiform and spiral bacilli of Vincent. Hess[2] states that (1) it is characterised by exudation on the tonsils, swelling of the glands in the neck, difficulty in swallowing, and fever, and has therefore a likeness to diphtheria, but runs a rapid and mild course; (2) it can be confounded with syphilis; (3) it is easily recognised by the bacteriological find; this consists in the most characteristic cases in a symbiosis of a bacillus and a spirillum. The clinical conditions are typical. On the first day one tonsil shows an easily detachable exudation, on the second day this membrane is found to rest on an ulcerated surface, and on the third and fourth days it becomes thicker and softer. In a few cases the membrane then becomes detached at its edges, and this is either coughed up or swallowed. The surface is slightly ulcerated. On it a new thin membrane forms, which is got rid of in the course of a few days. There is moderate fever and swelling of the cervical glands. These cases are termed "croupous," and only the fusiform bacillus is found. In the majority of cases the ulceration deepens under the membrane during the first four days. The pseudo-membrane is soft, grey, yellowish grey, or greenish in colour, and the surrounding tissue is reddened and œdematous. Swallowing becomes very

[2] *Deutsche med. Wchnschr.*, Oct. 15th, 1903; *Brit. M. J.*, 1904, i. Epitome, p. 1.

difficult; there is much salivation, and there is fetor *ex ore*. As a rule the constitutional changes are mild in comparison with the local condition, but these may also be severe. After about eight or ten days the membrane becomes detached, and the ulcerated surface soon gets clean. Complete removal of symptoms and signs rapidly follows, though the course of the disease may be prolonged.

The b. fusiformis is from 6 $\mu$ to 12 $\mu$ long; it is pointed at the ends, bulging in the centre. They are often arranged in pairs or diplobacilli, or in bundles, radiating, or at an acute angle to one another, or irregularly. They form vacuoles and other degenerated forms, but not spores. They are not stained by Weigert's or Gram's stain, but by fuchsine or methylene blue. They have long flagellæ, one at each end and two on each side. The spirillum is long and thin, does not stain by Gram, and only slightly by fuchsin. There are no flagellæ. Hess regards both as saprophytes of the mouth.

Two cases of ulcerative angina due to these bacilli are reported by Fisher,[1] who gives illustrations of the micro-organisms. One was clinically gangrenous tonsillitis. He mentions that the duration of the affection as described by Vincent, Bernheim, and others, varies from ten days to six weeks, and that there is a tendency to spontaneous cure. He finds no evidence that the disease is communicated easily from one individual to another.

**Tonsillitis, due to Suppurative Mammitis in Cows,** has been the cause of some recorded epidemics of sore throat. Thus Pierce[2] and Kenwood[3] have given interesting accounts of such outbreaks, and still more recently French[4] has described an outbreak at Finchley. French found that the disease was generally ushered in with chilliness and *malaise* about twenty-four hours after infection, and the throat felt sore. Its appearance was generally one of follicular tonsillitis or commencing quinsy. In no cases did any patches resemble diphtheria. In one or two instances the throat seemed to have escaped, and the infected milk produced an intense attack of diarrhœa. As the throat grew worse the glands of the neck became enlarged and matted together, and felt very hard and very painful. In rare instances suppuration occurred, but this was not the rule. At the same time the temperature rose to 103° or thereabouts. Neuralgia was a common symptom, and no nerve seemed exempt. One patient suffered from very acute neuralgia of the ophthalmic division of the fifth left cranial nerve, followed by herpes of those parts supplied by it. This was one of the cases complicated with erysipelas as hereinafter described. Other complications arose, such as œdematous swellings of the limbs, swellings of the

[1] *Am. J. M. Sc.*, 1903, cxxvi. 438.
[2] *Brit. M. J.*, 1903, ii. 1492. [3] *Ibid.*, 1904, i. 602. [4] *Ibid.*, p. 831.

joints resembling rheumatism, and occasionally erysipelas of the face and head, and a rash resembling measles on other cutaneous parts. A fatal termination was rare, and was generally due to some complication. In one instance in this epidemic cerebral intoxication producing Bell's mania proved fatal. A recrudescence of the symptoms was a very common factor.

Follicular tonsillitis, enlargement of the glands of the neck, and all other symptoms caused by this infected milk were not at all amenable to the usual remedies, and it was not until French used the anti-streptococcus serum (B. W. and Co.) that he noticed all symptoms abate, as well as the erysipelas.

With the same treatment in other severe cases the disease began to abate immediately, the temperature fell to normal within twelve hours, and the patients made rapid progress towards recovery. This treatment suggested that the cause of the disease was undoubtedly the streptococcus mammitis bovi, and Easte's bacteriological investigations bore out French's view, that the disease was that of "an erysipelatous infection."

It is contracted either by drinking the infected milk, or by contact with an infected patient.

**Treatment of Nasal Septal Deviations by Submucous Resection.**—Without entering into the relative merits of the numerous methods of correcting septal deviations and spurs, such as the Asche operation, the use of trephines, drills, electrolysis, &c., the excellent results following submucous resection deserve careful consideration in cases for which this somewhat recently practised method is suited. Cohn[1] considers that cases of displacement of the whole or greater part of the septum itself from the median line in the course of either the sagittal or horizontal axis, or in both planes, are peculiarly suitable for this method of operative treatment. Cohn's experience with the submucous resection dates from 1888, and he reminds us that it is really to Krieg, of Stuttgart, that the credit belongs of having most systematically described the method in 1886, though it had been recommended by Hartmann in 1883. Thus the method is not novel; but it is not generally known as a well-tried procedure, hence the remarks of one who has had a large experience in submucous resection are exceedingly valuable, and are quoted at length. In the earlier cases the operation was usually performed under general anæsthesia. Later on, since the introduction of adrenalin, a combination of a weak cocaine solution and adrenalin has usually sufficed in most cases. Instead of employing the sitting posture, as recommended by Krieg, Cohn usually places the patient on the table on his back with the head slightly raised and the chin depressed, the head slightly turned away from the deviated side. The

[1] *Med. Rec.*, 1903, lxiv. 1013.

nostrils being held back by retractors, an incision slightly convex is made through the whole length of the mucous membrane as near to the point of greatest convexity as possible and only through the mucous membrane. Care in not cutting through the cartilage at the first incision is of value in enabling a more rapid separation of the mucous membrane from the cartilage. The mucous membrane is then held back by a small sharp hook, and the cartilage, after being exposed as far as possible, is nicked and then gradually separated from the mucous membrane on the other side. After the cartilage has been freed as far as possible, especial attention being paid not to dislocate it, an incision above is made parallel to the ridge of the nose in order to prevent an external deformity, a second incision made below, and the cartilage finally dissected out posteriorly; the first piece removed usually embraces the greater portion of the protuberant cartilage. The still protruding pieces must be removed piece by piece, working from before backward, and, if necessary, even removing the bony portion with a small bone forceps. An occasional revision and replacing of the mucous membrane is the best guide as to the amount which must be removed. In suitable cases, with large noses and anterior deviations, the operation may be finished rapidly, in some cases twenty minutes sufficing for the whole operation; in more difficult cases the operation may take as long as two to two and a half hours. After the cartilage has been removed the mucous membrane is carefully replaced; if it has been torn and forms a flap, one or two sutures may be added to keep it in place. The nares are then lightly packed with gauze and the patient kept in bed for a day or two. Ice applications over the nose and change of dressing every twenty-four hours for two or three days is usually the only treatment required. After that the patient may be discharged, no further *after-treatment* being necessary except the use of an ointment or an oil spray to prevent formation of crusts until the mucous membrane has regained its normal condition.

P. WATSON WILLIAMS.

